# Iron deficiency in *JAK2* exon12 and *JAK2-*V617F mutated polycythemia vera

**DOI:** 10.1038/s41408-021-00552-x

**Published:** 2021-09-17

**Authors:** Dan Liu, Zefeng Xu, Peihong Zhang, Tiejun Qin, Bing Li, Shiqiang Qu, Lijuan Pan, Wenyu Cai, Jinqin Liu, Huijun Wang, Qi Sun, Xiujuan Sun, Meng Jiao, Qingyan Gao, Zhongxun Shi, Huijun Huang, Gang Huang, Robert Peter Gale, Zhijian Xiao

**Affiliations:** 1 State Key Laboratory of Experimental Hematology, National Clinical Research Center for Blood Diseases, Institute of Hematology and Blood Diseases Hospital, Chinese Academy of Medical Sciences & Peking Union Medical College, Tianjin, China; 2 MDS and MPN Centre, Institute of Hematology and Blood Diseases Hospital, Chinese Academy of Medical Sciences & Peking Union Medical College, Tianjin, China; 3 Hematologic Pathology Center, Institute of Hematology and Blood Diseases Hospital, Chinese Academy of Medical Sciences & Peking Union Medical College, Tianjin, China; 4 Divisions of Experimental Hematology and Cancer Biology, Cincinnati Childrens Hospital Medical Center, Cincinnati, OH USA; 5 Haematology Research Centre, Department of Immunology and Inflammation, Imperial College London, London, UK

**Keywords:** Cancer metabolism, Myeloproliferative disease

**Dear Editor**,

Somatic driver mutations in *JAK2* (*JAK2*^V617F^ and exon 12 mutations) are detected >95% of persons with polycythemia vera (PV) [1–4]. Iron deficiency is universal in persons with PV at diagnosis and can be worsened by phlebotomy [5]. Precise mechanisms of iron deficiency in persons with PV at diagnosis are unknown. A previous study reported heterogeneous bone marrow expression of erythroferrone (ERFE) and hepcidin, important regulators of iron metabolism, in mice with *JAK2*^V617F^ or *JAK2*^exon12^ mutation [6].

The relationship between iron deficiency and the type of *JAK2* mutations in persons with PV is unknown. We studied this issue in 305 subjects who were >18 years old with newly diagnosed PV seen at Blood Diseases Hospital, Chinese Academy of Medical Sciences from June 1, 2007 to February 28, 2020. Diagnosis of PV was based on the 2016 World Health Organization (WHO) criteria [7]. All subjects provided informed consent in compliance with the Declaration of Helsinki.

The median age was 59 years (IQR, 49–66 years). 158 (52%) were men. 11 (5%) of 228 subjects had abnormal diagnosis cytogenetics. 293 (96%) had *JAK2*^V617F^ and 12 (4%), a *JAK2*^exon12^ mutation. The median *JAK2*^V617F^ variant allele frequency (VAF) was 54% (IQR, 33–73%). Subjects with a *JAK2*^exon12^ mutation had higher RBC concentrations (medians, 8.60 versus 7.11 × 10^12^/L; *p* < 0.001) and hematocrits (medians, 64.7% versus 59.7%; *p* = 0.002) compared with subjects with *JAK2*^V617F^ but lower concentrations of WBC (medians, 8.75 versus 12.95 × 10^9^/L; *p* = 0.005), platelet (medians, 273 versus 474 × 10^9^/L; *p* = 0.011) and serum erythropoietin (EPO) (medians, 0.68 versus 1.16 mIU/L; *p* = 0.005), which were consistent with previous studies [8–10]. There was no significant difference in hemoglobin concentration (medians, 194 g/L versus 194 g/L; *p* = 0.616).

Subjects with transferrin saturation (TSAT) <20% or ≥20% were defined as iron-deficient and iron-sufficient, respectively [11]. 159 (52%) were iron deficient at diagnosis. Detail clinical and laboratory co-variates of subjects with iron deficiency are displayed in Table 1. Subjects with iron deficiency had higher concentrations of RBCs (medians, 7.51 versus 6.67 × 10^12^/L; *p* < 0.001) and hematocrits (medians, 60.9% versus 58.9%; *p* = 0.003) and lower serum EPO concentrations (medians, 1.07 versus 1.35 mIU/mL; *p* = 0.021) compared with subjects, not iron deficient. There were no significant differences in diagnosis hemoglobin, WBC or platelet concentrations (*p* > 0.05; Table 1).

**Table 1:** Co-variates of PV patients with and without TSAT < 20% at diagnosis.

Variables	TSAT < 20% (*n* = 159)	TSAT ≥ 20% (*n* = 146)	*p*
Female, *n* (%)	86 (54%)	61 (42%)	0.032
Age, *n* (%)	60 (24–86)	58 (27–84)	0.217
RBC, ×10^12^/L; median (range)	7.51 (5.48–10.94)	6.67 (4.65–9.39)	<0.001
Hemoglobin, g/L; median (range)	193 (150–241)	195 (157–245)	0.855
Hematocrit, %; median (range)	60.9 (49.3–79.0)	58.9 (47.2–74.3)	0.003
WBC, ×10^9^/L; median (range)	13.95 (3.97–45.59)	12.49 (3.75–34.28)	0.350
Platelets, ×10^9^/L; median (range)	458 (127–1866)	506 (65–1609)	0.134
MCV, fL; median (range)	80.4 (61.4–100.4)	87.5 (75.7–106.2)	<0.001
MCH, pg; median (range)	25.7 (16.0–34.0)	29.0 (23.3–36.8)	<0.001
MCHC, g/L; median (range)	318 (248–354)	331 (299–365)	<0.001
*JAK2*^V617F^ VAF, %; median (range) (*N* = 229)^a^	61 (6–92)	47 (5–91)	0.003
*JAK2*^exon12^ mutation, *n* (%)	11 (7%)	1 (1%)	0.006
EPO, mIU/mL; median (range) (*N* = 194)	1.07 (0.08–5.02)	1.35 (0.40–7.21)	0.021
Abnormal cytogenetics, *n* (%) (*N* = 228)	5/122 (4%)	6/106 (6%)	0.759

*PV* polycythemia vera, *TSAT* transferrin saturation, RBC red blood cell, *MCV* mean corpuscular volume, *MCH* mean corpuscular hemoglobin, *MCHC* mean corpuscular hemoglobin concentration, *VAF* variant allele frequency, *EPO* erythropoietin, *UIBC* unsaturated iron-binding capacity, *TIBC* total iron-binding capacity.

^a^In *JAK2*^V617F^ mutated patients.

The severity of iron deficiency differed based on *JAK2* mutation types. Subjects with a *JAK2*^exon12^ mutation were more likely to be iron deficient (92% versus 51%; *p* = 0.006) and had lower serum iron (medians, 5.1 versus 11.9 μmol/L; *p* = 0.002) and ferritin concentrations (medians, 13.9 versus 32.2 ng/mL; *p* = 0.004) compared with subjects with *JAK2*^V617F^ (Fig. 1A). Declined mean corpuscular volume (MCV), mean corpuscular hemoglobin (MCH), and mean corpuscular hemoglobin concentration (MCHC) were significantly more frequent in subjects with *JAK2*^exon12^ mutation (*p* < 0.05), consistent with their more severe iron deficiency (Fig. 1B). *JAK2*^exon12^ mutation was an independent factor associated with iron deficiency by multivariable analysis that adjusted by age and sex (HR = 11.185, 95% confidence interval [CI] 1.404–89.089, *p* = 0.023; Supplementary Table 1).

**Fig. 1:**
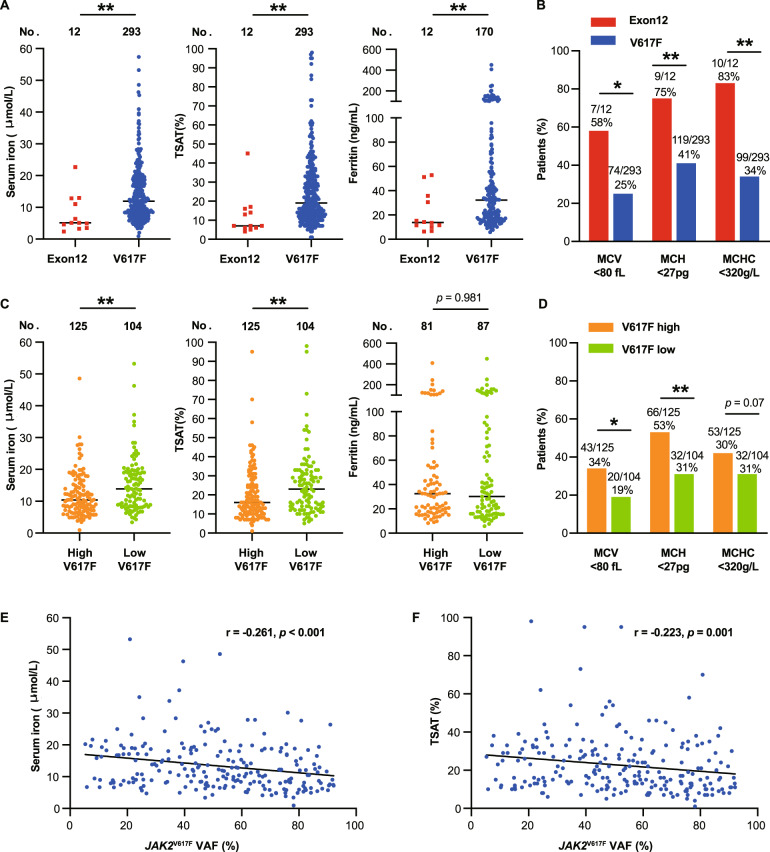
The relationship between iron deficiency and JAK2 mutations in PV patients. **A** Serum iron, TSAT, and ferritin were significantly lower in *JAK2*^exon12^ mutated patients than in *JAK2*^V617F^ mutated patients. **B** Declined MCV, MCH, MCHC was more frequent in *JAK2*^exon12^ mutated patients compared with *JAK2*^V617F^ mutated patients. **C** Serum iron, TSAT were significantly lower in patients with low *JAK2*^V617F^VAF (<50%) than in patients with high *JAK2*^V617F^ VAF (≥50%) among *JAK2*^V617F^ mutated patients, but the ferritins were comparable between two these two cohorts. **D** Declined MCV, MCH, MCHC was more frequent in subjects with high *JAK2* ^V617F^ VAFs compared with low *JAK2*^V617F^ VAFs. **E** and **F** *JAK2*^V617F^ VAF negatively correlated with serum iron and TAST in *JAK2*^V617F^ mutated patients. PV polycythemia vera, TSAT transferrin saturation, MCV mean corpuscular volume, MCH mean corpuscular hemoglobin, MCHC mean corpuscular hemoglobin concentration, VAF variant allele frequency. *p < 0.05; **p < 0.01; ***p < 0.001.

The severity of iron deficiency is also correlated with the *JAK2*^V617F^ allele burden. Subjects with *JAK2*^V617F^ VAFs ≥ 50% were more likely to be iron deficient (61% versus 44%, *p* = 0.013) and had lower serum iron (medians, 10.4 versus 13.7 μmol/L; *p* = 0.002) compared with subjects with *JAK2*^V617F^ VAFs < 50% (Fig. 1C). *JAK2*^V617F^ VAF was weakly negatively correlated with iron deficiency (Fig. 1E, F). *JAK2*^V617F^ VAF was significantly higher in iron-deficient subjects (medians, 61% versus 47%; *p* = 0.003; Table 1). Consistently, declined MCVs, MCHs, and MCHCs were more frequent in subjects with *JAK2*^V617F^ VAFs ≥ 50% (Fig. 1D). *JAK2*^V617F^ VAF > 50% was an independent factor associated with iron deficiency by multivariable analysis that adjusted by age and sex (HR = 2.022, 95% CI 1.186–3.447, *p* = 0.010; Supplementary Table 2).

Before the publication of the 2016 WHO diagnostic criteria of PV, there were people defined as *masked* PV with *JAK2*^V617F^ or *JAK2*^exon12^ mutations and with bone marrow histological features of PV, but not meeting hemoglobin concentration or hematocrit threshold defined in the World Health Organization (WHO) or British Criteria for Standards in Hematology (BCSH) PV diagnostic criteria [12, 13]. These low values were likely the result of iron deficiency [12, 13]. As discussed above, hematocrits were higher in subjects with iron deficiency compared with those without iron deficiency inconsistent with their comparable hemoglobin concentrations. Consequently, we compared the diagnostic accuracy of these co-variates according to 2016 WHO diagnostic criteria in subjects with and without iron deficiency stratified for sex [7]. We stratified subjects by sex because females are more often iron deficient because of menstruation. In the iron-deficient cohort, all subjects met the hematocrit thresholds for PV as defined in the 2016 WHO diagnostic criteria [7], but there were 7% of subjects not meeting the threshold of hemoglobin concentration for both sexes (Supplementary Fig. 1A, D). These data indicate hematocrit is more sensitive than hemoglobin concentration as an indicator of PV in persons who are iron deficient (sensitivities, 100% [86/86] versus 93% [80/86], *p* = 0.013 for females; 100% [73/73] versus 93% [68/73], *p* = 0.023 for males; Supplementary Fig. 1C, F). But in subjects without iron deficiency, there were comparable percentages of subjects not meeting the diagnostic threshold of HCT or HB (Supplementary Fig. 1B, E), and the diagnostic sensitivities were not significantly different between HCT and HB (97% [59/61] versus 98% [60/61], *p* = 0.559 for females; 94% [80/85] versus 97% [82/85], *p* = 0.469 for males; Supplementary Fig. 1C, D). We also found the diagnostic sensitivity of hematocrit was superior to hemoglobin concentration in males with a *JAK2*^V617F^ VAF ≥ 50% (98% [66/67] versus 91% [61/67], *p* = 0.052; Supplement Fig. 2F).

44 (14%) subjects lost follow-up. Among the remaining patients, the median follow-up for subjects with or without iron deficiency was 35 months (IQR, 15–68 months) and 36 months (IQR, 20–69), respectively. The 5-year accumulative incidence of death and thrombotic events were not significantly different between subjects with or without iron deficiency (4% versus 11%, *p* = 0.233; 3% versus 6%, *p* = 0.389; Supplementary Fig. 3).

Our study has limitations. For example, this is a retrospective study from our single-center, and data of iron metabolism were available in part of newly diagnosed subjects in our center.

In summary, as far as we know, this is the first report of the relationship between iron deficiency and type of *JAK2* mutations in patients with PV in the English literature until now, our data showed that iron deficiency is more common in subjects with PV and *JAK2*^exon12^ mutation compared with those with *JAK2*^V617F^, and *JAK2*^V617F^ allele burden correlates with the probability of iron deficiency. Also, hematocrit was more sensitive than hemoglobin concentration as a basis to diagnosis PV in persons with iron deficiency. Regardless of these data, the mechanism(s) by which *JAK2* mutations affect iron metabolism needs further study.

## Supplementary information

Supplementary tables.

Supplementary Figure 1.

Supplementary Figure 2.

Supplementary Figure 3.
