# Supplementary material for: Iron deficiency in JAK2 exon12 and JAK2-V617F mutated polycythemia vera
Source: Blood Cancer J. 2021 Sep 17;11(9):154. doi: 10.1038/s41408-021-00552-x (PMC8448748; doi:10.1038/s41408-021-00552-x)
Supplement: Supplementary file 1 — Supplementary tables. [file 41408_2021_552_MOESM1_ESM.docx]

**Supplementary table 1. Uni- and multi-variable analysis of sex, age and JAK2 mutation types in possibility of iron deficiency.**

| Variables | Univariable | | | Multivariable | | |
| --- | --- | --- | --- | --- | --- | --- |
|  | HR | 95%CI | P-value | HR | 95%CI | P-value |
| Female | 1.642 | 1.043-2.583 | 0.032 | 1.688 | 1.061-2.687 | **0.027** |
| Age > 62 years | 1.495 | 0.935-2.390 | 0.093 | 1.605 | 0.991-2.598 | 0.054 |
| *JAK2*^exon12^ mutation | 10.777 | 1.374-84.547 | 0.024 | 11.185 | 1.404-89.089 | **0.023** |

*:Cut-offs of age was defined by receiver operator characteristic (ROC) curve.

**Supplementary table 2. Uni- and multi-variable analysis of sex, age and JAK2^V617F^ allele burden in possibility of iron deficiency.**

| Variables | Univariable | | | Multivariable | | |
| --- | --- | --- | --- | --- | --- | --- |
|  | HR | 95%CI | P-value | HR | 95%CI | P-value |
| Female | 1.642 | 1.043-2.583 | 0.032 | 1.448 | 0.848-2.472 | 0.175 |
| Age > 62 years* | 1.495 | 0.935-2.390 | 0.093 | 1.347 | 0.784-2.317 | 0.281 |
| *JAK2*^V617F^ VAF > 50% | 2.034 | 1.199-3.450 | 0.008 | 2.022 | 1.186-3.447 | **0.010** |

*:Cut-offs of age was defined by receiver operator characteristic (ROC) curve.
