# Supplementary material for: Iron deficiency in JAK2 exon12 and JAK2-V617F mutated polycythemia vera
Source: Blood Cancer J. 2021 Sep 17;11(9):154. doi: 10.1038/s41408-021-00552-x (PMC8448748; doi:10.1038/s41408-021-00552-x)
Supplement: Supplementary file 2 — Supplementary Figure 1. [file 41408_2021_552_MOESM2_ESM.pdf]

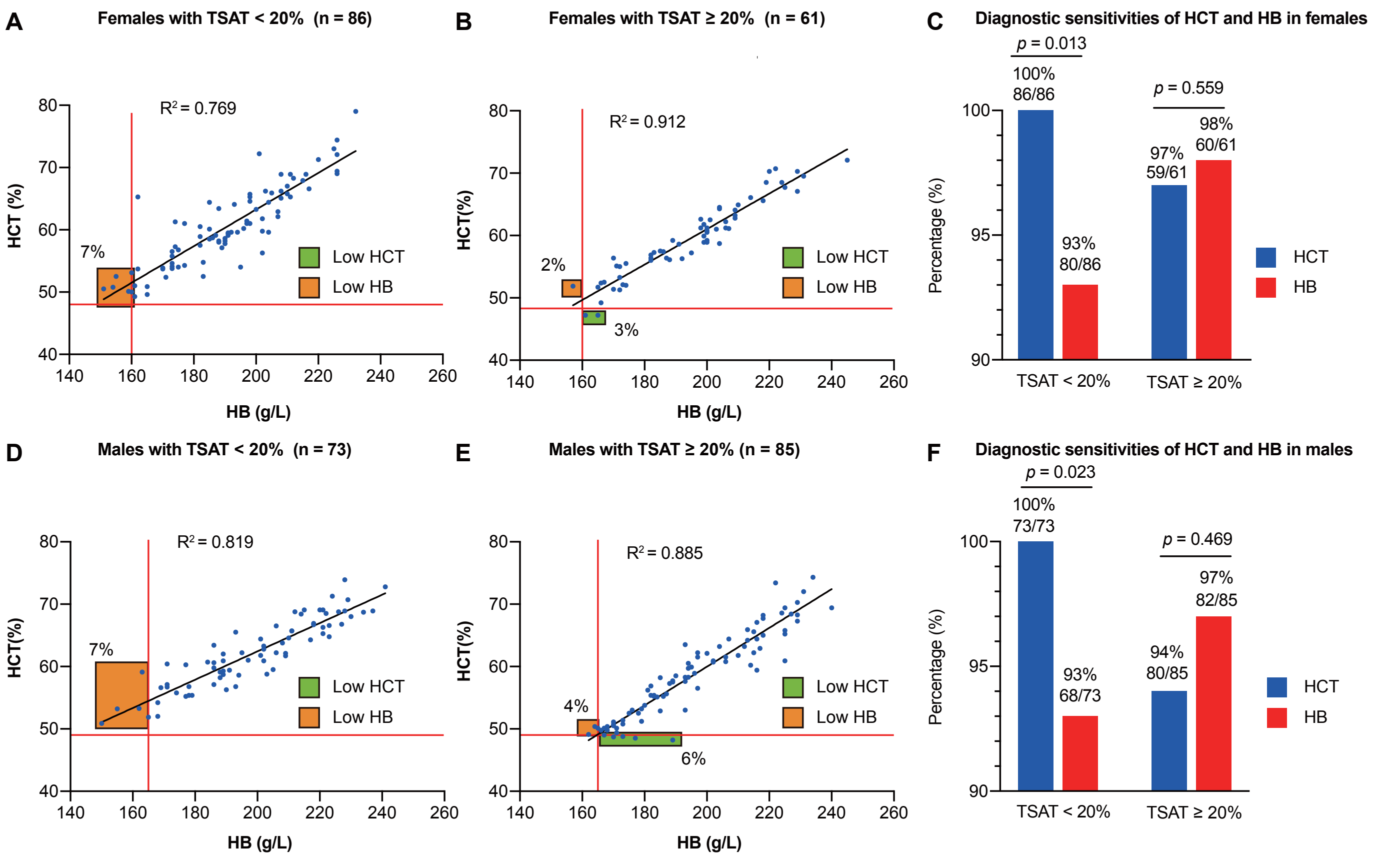

Supplementary Figure 1. Diagnostic sensitivity of hemoglobin and hematocrit for PV with or without iron deficiency.

The percentages of patients with hemoglobin or hematocrit lower than the threshold defined in 2016 WHO diagnostic criteria in patients with iron deficiency (**A** and **D**) and without iron deficiency (**B** and **E**) for females and males. Diagnostic sensitivities of HCT and HB in females (**C**) and males (**F**) with or without iron-deficiency.

PV: polycythemia vera; TSAT: Transferrin saturation ; R: spearman rank correlation coefficient; HB: hemoglobin; HCT: hematocrit; Low HB: HB≤160g/L in female or ≤165g/L in male; low HCT: HCT≤48% in female or ≤49% in male.
