# Supplementary material for: Iron deficiency in JAK2 exon12 and JAK2-V617F mutated polycythemia vera
Source: Blood Cancer J. 2021 Sep 17;11(9):154. doi: 10.1038/s41408-021-00552-x (PMC8448748; doi:10.1038/s41408-021-00552-x)
Supplement: Supplementary file 3 — Supplementary Figure 2. [file 41408_2021_552_MOESM3_ESM.pdf]

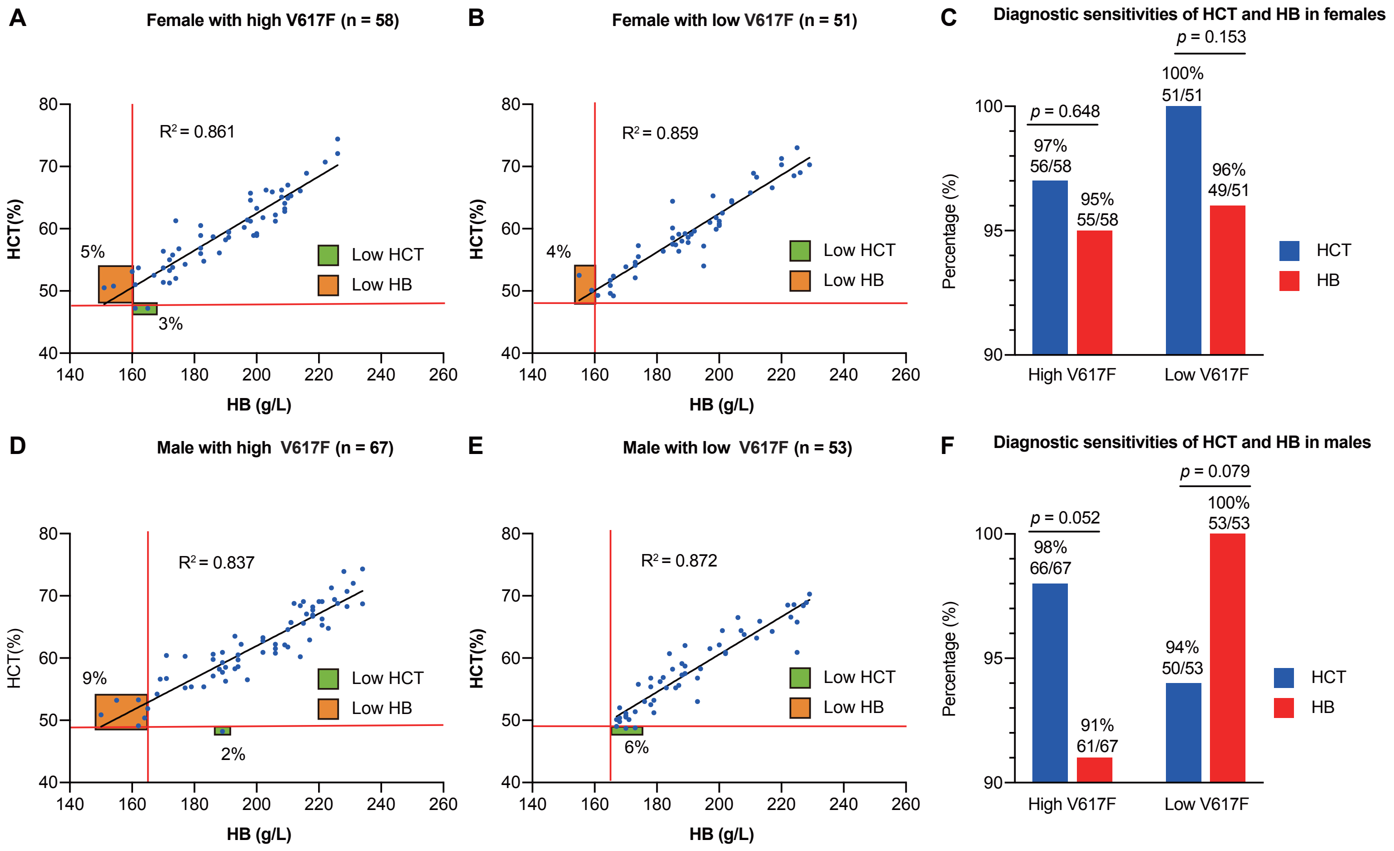

Supplementary Figure 2. Diagnostic sensitivity of hemoglobin and hematocrit for PV patients with different *JAK2*^{V617F} allele burdens.

The percentages of patients with hemoglobin or hematocrit lower than the threshold defined in 2016 WHO diagnostic criteria in patients with high (≥50%; **A** and **D**) and low (<50%; **B** and **E**) *JAK2*^{V617F} VAF for females and males. Diagnostic sensitivities of HCT and HB in females (**C**) and males (**F**) with high (≥50%) and low (≥50%) *JAK2*^{V617F} VAF.

PV: polycythemia vera; R: spearman rank correlation coefficient; HB: hemoglobin; HCT: hematocrit; VAF: variant allele frequency. Low HB: HB≤160g/L in female or ≤165g/L in male; low HCT: HCT≤48% in female or ≤49% in male.
