# Supplementary material for: Iron deficiency in JAK2 exon12 and JAK2-V617F mutated polycythemia vera
Source: Blood Cancer J. 2021 Sep 17;11(9):154. doi: 10.1038/s41408-021-00552-x (PMC8448748; doi:10.1038/s41408-021-00552-x)
Supplement: Supplementary file 4 — Supplementary Figure 3. [file 41408_2021_552_MOESM4_ESM.pdf]

A

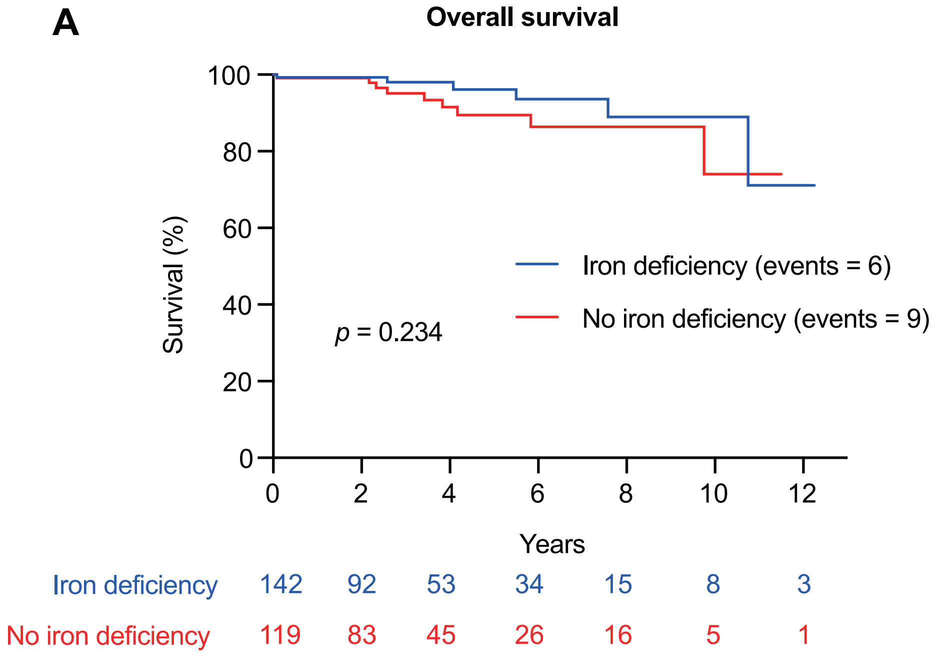

B

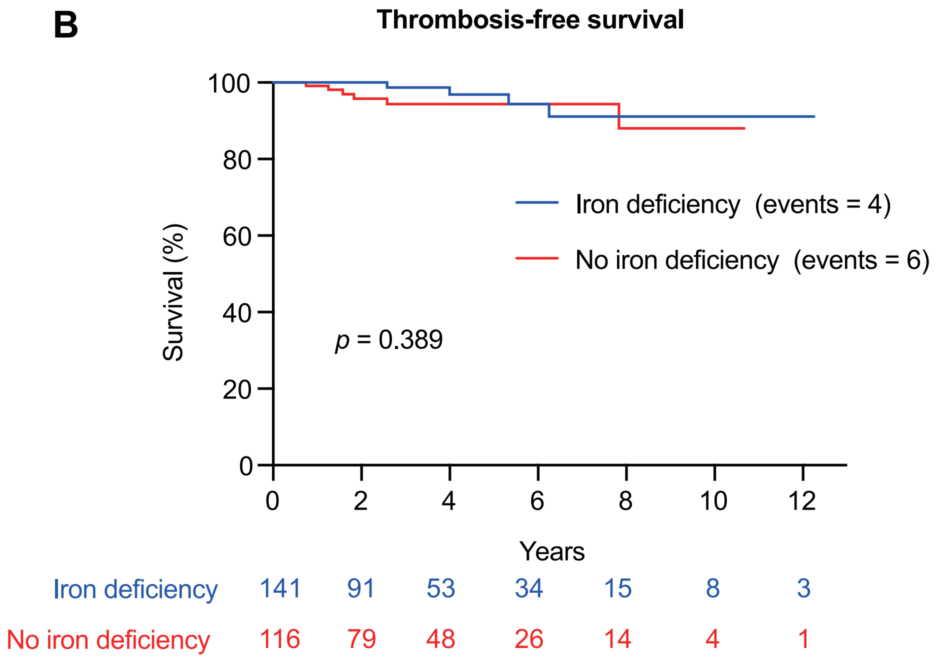

Supplementary Figure 3. Survivals for subjects with or without iron deficiency.
Overall survivals (**A**) and thrombosis-free survivals (**B**) for subjects with or without iron deficiency. 4 subjects died without details of thrombosis events.
